# Delayed diagnosis of hereditary fructose intolerance presenting as chronic lean steatosis in an adolescent

**DOI:** 10.1002/jpr3.70197

**Published:** 2026-07-13

**Authors:** Alexandra Hurlock, Melissa Lah, Hannah Sue Hyaduck, Jean P. Molleston, Chaowapong Jarasvaraparn

**Affiliations:** ^1^ Indiana University School of Medicine Indianapolis Indiana USA; ^2^ Department of Medical and Molecular Genetics Indiana University School of Medicine/Riley Hospital for Children Indianapolis Indiana USA; ^3^ Division of Pathology Indiana University School of Medicine/Riley Hospital for Children Indianapolis Indiana USA; ^4^ Division of Pediatric Gastroenterology, Hepatology and Nutrition Indiana University School of Medicine/Riley Hospital for Children Indianapolis Indiana USA

**Keywords:** ALDOB gene mutation, aldolase B deficiency, children, metabolic dysfunction‐associated steatotic liver disease (MASLD)

## Abstract

Hereditary fructose intolerance (HFI) typically presents in infancy with acute metabolic crisis upon the introduction of fructose. We report a case of a 13‐year‐old female with chronic abdominal pain, short stature, and persistent mild transaminitis. Despite a late presentation, hepatic steatosis was identified on ultrasound and biopsy. Genetic testing confirmed a homozygous pathogenic ALDOB variant. This case illustrates that self‐imposed dietary avoidance can mask classic HFI symptoms, leading to a delayed diagnosis of “lean” hepatic steatosis in adolescence.

## 1 INTRODUCTION

Hereditary fructose intolerance (HFI) is a rare autosomal recessive disorder resulting in the deficiency of aldolase B, the enzyme responsible for metabolism of fructose‐1‐phosphate. Accumulation of fructose‐1‐phosphate is hepatotoxic, resulting in macrovesicular steatosis. Presentation of HFI occurs in infancy, after the introduction of fructose‐containing foods, with nausea, vomiting, and failure to thrive precipitated by the underlying metabolic derangements such as hypoglycemia, lactic acidosis, and electrolyte disturbances. Untreated individuals may develop progressive hepatic and renal disease leading to organ failure if treatment through diet modification and nutrition supplementation is not initiated.^1^ Nonclassical HFI has been documented in pediatric patients exhibiting failure to thrive, global developmental delay, and short stature. In adults, clinical signs may include hypothalamic amenorrhea alongside a lifelong aversion to sweets.^2^ There is a high prevalence of steatosis in HFI patients that is not related to obesity or insulin resistance.^3^, ^4^ We describe a case of delayed diagnosis in a 13‐year‐old presenting with chronic non‐specific symptoms and lean steatosis.

## 2 CASE REPORT

A 13‐year‐old female was referred to Pediatric Gastroenterology for persistent mild elevation of liver enzymes, nausea, and abdominal pain without jaundice for 2 years. Her aspartate aminotransferase (AST) and alanine aminotransferase (ALT) were minimally elevated to 40–50 IU/L. She had a lifelong history of short stature, falling below the third percentile, and a body mass index (BMI) of 23–24.9 (*z* score 0.99–1.1) with accelerated weight gain over the last few years. Further workup with Endocrinology revealed a normal bone age with near closure of growth plates with height below the third percentile, thought to be from familial short stature. A comprehensive evaluation for Wilson disease, α1‐antitrypsin deficiency, celiac disease, lysosomal storage disorders, autoimmune hepatitis, and viral hepatitis C was negative. Abdominal ultrasound revealed hepatic steatosis, while FibroScan showed a normal liver stiffness measurement (LSM) of 6.1 kPa with 5% interquartile range. The patient did not have a controlled attenuation parameter (CAP) score from FibroScan due to a small probe.

**Figure 1:**
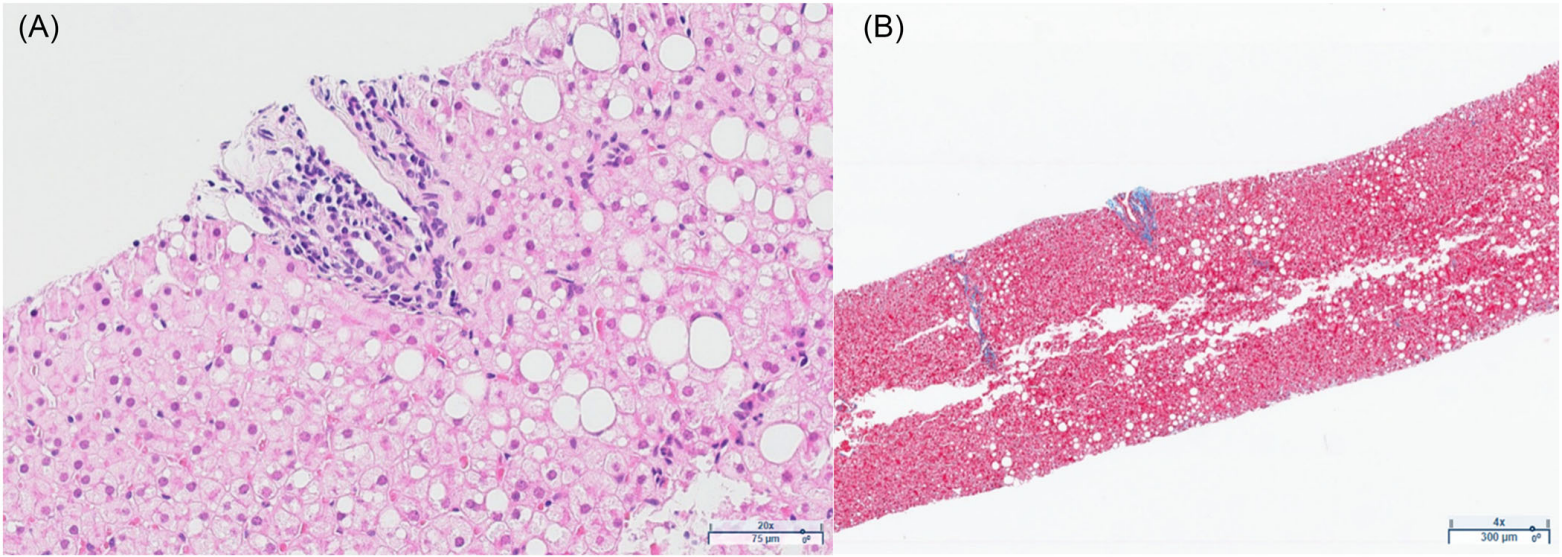
Hematoxylin and eosin and trichrome stains of liver biopsy. (A) Liver core biopsy showing mild macrovesicular steatosis (20%–30% of hepatocytes). (B) A trichrome stain shows normal architecture and no increased fibrosis.

Due to persistent steatosis of unknown etiology, a liver biopsy was performed. Histology demonstrated 20%–30% macrovesicular steatosis without inflammation or fibrosis (Figure 1). Given the “lean” steatosis phenotype, a genetic cholestasis and metabolic panel was ordered. Genetic analysis identified a homozygous pathogenic variant in ALDOB (c.448G>C; p.Ala150Pro), confirming the diagnosis of HFI. This specific variant is the most prevalent globally, accounting for approximately 53%–68% of HFI alleles in Western populations.^5^, ^6^

She was referred to Metabolism Clinic, where further pertinent HFI history was obtained. The patient started having abdominal pain and poor weight gain at age 6 years but did not follow up with Gastroenterology when referred. At 12‐years‐old, she began having periods of dizziness, shakiness, and changes in her vision when she ate fruit or consumed sugary foods or beverages. Her diet consisted of frequent fast food, chicken, pork, few vegetables, and 2–3 gallons of milk per week. Throughout her life, she had avoided fruits, desserts, juice, and other products high in fructose due to the abdominal pain, dizziness, and loss of vision she experienced after consumption. Following diagnosis, she was counseled on strict avoidance of fructose, sucrose, sorbitol, and polysorbate, with glucose monitoring for symptoms.

The patient continues to have symptoms every week or every few weeks with shakiness, dizziness, and occasional vomiting. She adheres to her recommended diet and monitors her blood sugars, treating low blood sugars with glucose tabs without evidence of hypoglycemia. After 6 months of diet restriction, she had a repeat abdominal ultrasound revealing resolution of hepatic steatosis with still normal LSM at 4.4 kPa from FibroScan. Her latest AST and ALT have normalized to 18 and 17 U/L, respectively. With near growth plate closure at presentation, our patient unsurprisingly remains right below the third percentile for height. She remains under gastroenterology and metabolism care. We summarized the clinical timeline in Table 1.

**Table 1:** A timeline outlining this patient's presentation and subsequent workup throughout the years.

Years	Month^a^	Clinical timeline
2009		Patient is born
2015–2016		Abdominal pain and poor weight gain, referred to GI. No follow‐up appointment occurred
2021–2022		Patient has episodes of dizziness, shaking, and changes in vision
2022		Primary care physician seen for nausea not associated with pain. Found to have elevated alkaline phosphatase, AST, and ALT
2022	December	Found to have fatty infiltration of the liver and small gallbladder polyp
2023	July	Repeat US, AST, and ALT persistently elevated; referred to our pediatric GI
	August	Pediatric GI visit; steatosis workup negative.
	August	Liver US displays diffuse parenchymal echogenicity suggestive of hepatic steatosis
	October	Seen by pediatric endocrinology for short stature; near growth plate closure
	December	Initial FibroScan, which showed normal liver stiffness measurement
2024	March	Liver biopsy showing macrovesicular steatosis
	April	Cholestasis genetic panel sent out
2025	April	Genetic panel results: homozygous pathogenic variant in ALDOB
	June	Pediatric genetics and metabolism initial appointment and diet changes implemented
	November	Repeat US with no hepatic steatosis, repeat FibroScan, and normalized AST and ALT

Abbreviations: ALT, alanine aminotransferase; AST, aspartate aminotransferase; GI, gastroenterology; US, ultrasound.

^a^ If applicable.

## 3 DISCUSSION

HFI is a rare autosomal recessive enzymatic deficiency that affects 1 in 20,000 live births in the United States. While the classic presentation involves acute hypoglycemia and lactic acidosis in infants, this case highlights a more indolent, “masked” progression.^1^ The patient's survival into adolescence without significant hepatic or renal failure was likely due to her autonomous, symptom‐driven avoidance of high‐fructose foods—a phenomenon often reported in undiagnosed HFI patients who develop a “natural” distaste for sweets. Despite this, the chronic low‐level fructose intake from hidden sources was sufficient to cause persistent macrovesicular steatosis and growth retardation.

This case underscores two critical clinical points:

1. HFI as a differential for “lean steatosis”: In patients with hepatic steatosis and a low or normal BMI, HFI should be considered even in the absence of acute metabolic crises.
2. In the setting of unexplained elevated liver enzymes and steatosis in lean children, one should be prompted to complete an investigation into inborn errors of metabolism and pursue genetic testing.

The clinical significance of trace fructose intake is highlighted by Di Dato et al.,^7^ who observed that 94% of children with HFI had ultrasound evidence of steatosis. Interestingly, 38% maintained high ALT levels regardless of the specific quantity of residual fructose consumed. This supports the observation in our case that partial dietary avoidance is often insufficient to prevent progressive hepatic steatosis and biochemical injury.

## 4 CONCLUSION

HFI can remain undiagnosed until adolescence. Clinicians should maintain a high index of suspicion for HFI in patients with unexplained lean steatosis and non‐specific gastrointestinal symptoms, particularly when a history of self‐selected dietary restrictions is present. Early genetic testing can prevent invasive procedures and lead to rapid clinical improvement through dietary management.

## CONFLICT OF INTEREST STATEMENT

The authors declare no conflicts of interest.

## ETHICS STATEMENT

Informed consent was obtained from the patient's parents.
